# Effectiveness of mobile robots collecting vital signs and radiation dose rate for patients receiving Iodine-131 radiotherapy: A randomized clinical trial

**DOI:** 10.3389/fpubh.2022.1042604

**Published:** 2023-01-09

**Authors:** Dan Li, Dingwei Gao, Suyun Fan, GangHua Lu, Wen Jiang, Xueyu Yuan, Yanyan Jia, Ming Sun, Jianjun Liu, Zairong Gao, Zhongwei Lv

**Affiliations:** ^1^Department of Nuclear Medicine, Shanghai Tenth People's Hospital, School of Medicine, Tongji University, Shanghai, China; ^2^Department of Nuclear Medicine, Sun Yat-sen Memorial Hospital, Sun Yat-sen University, Guangzhou, Guangdong, China; ^3^Shanghai Tenth People's Hospital, Tongji University, Shanghai, China; ^4^Department of Nuclear Medicine, Renji Hospital, School of Medicine, Shanghai Jiao Tong University, Shanghai, China; ^5^Department of Nuclear Medicine, Union Hospital, Tongji Medical College, Huazhong University of Science and Technology, Wuhan, Hubei, China

**Keywords:** mobile robot, 131I treatment, radiation safety, vital signs and radiation dose rate, effectiveness

## Abstract

**Objective:**

Patients receiving radionuclide 131I treatment expose radiation to others, and there was no clinical trial to verify the effectiveness and safety of mobile robots in radionuclide 131I isolation wards. The objective of this randomized clinical trial was to evaluate the effectiveness and safety of mobile robots in providing vital signs (body temperature and blood pressure) and radiation dose rate monitoring for patients receiving radionuclide therapy.

**Methods:**

An open-label, multicenter, paired, randomized clinical trial was performed at three medical centers in Shanghai and Wuhan, China, from 1 April 2018 to 1 September 2018. A total of 72 participants were assigned to the group in which vital signs and radiation doses were both measured by mobile robots and conventional instruments. Intergroup consistency, completion rate, and first success rate were the primary effectiveness measures, and vital sign measurement results, the error rate of use, and subjective satisfaction were secondary indicators. Adverse events related to the robot were used to assess safety.

**Results:**

Of the 72 randomized participants (median age, 39.5; 27 [37.5%] male participants), 72 (100.0%) completed the trial. The analysis sets of full analysis set, per-protocol set, and safety analysis set included 72 cases (32 cases in Center A, 16 cases in Center B, and 24 cases in Center C). The consistency, completion rate, and first success rate were 100% (*P* = 1.00), and the first success rates of vital signs and radiation dose rate were 91.7% (*P* = 1.000), 100.0% (*P* = 0.120), and 100.0% (*P* = 1.000). There was no significant difference in vital signs and radiation dose rate measurement results between the robot measurement group and the control group (*P* = 0.000, 0.044, and 0.023), and subjective satisfaction in the robot measurement group was 71/72 (98.6%), compared to 67/72 (93.1%) in the control group. For safety evaluation, there was no adverse event related to the mobile robot.

**Conclusion:**

The mobile robots have good effectiveness and safety in providing vital signs and radiation dose rate measurement services for patients treated with radionuclides.

## 1. Introduction

Radionuclide therapy plays an increasingly important role in treating a range of cancers in the last few years; we have witnessed unprecedented advances in the field of nuclear medicine (1, 2). Significantly, one of the main driving forces is the so-called theranostic concept that combines the diagnostic use of one biomarker with a therapeutic option. Some studies have confirmed the value of combined radionuclide PET/CT in neuroendocrine tumors (3), prostate cancer (4), or hematological malignancies (5, 6) for the evaluation of efficacy, metastasis, or recurrence monitoring, and some tumors could even be treated with radionuclide-labeled antibodies. Especially, thyroid treatment post-thyroidectomy is the classical protocol where sufficiently large activities of radionuclide 131I are given by mouth to the patient (7); it is still a unique method of cancer treatment (8–11). Iodine-131 (131I) therapy as one of the post-surgical targeted therapies has been proven as an effective treatment modality in reducing the risk of recurrence and mortality in intermediate- and high-risk differentiated thyroid cancer (DTC) (12).

Patients receiving 131I treatment after thyroidectomy exposes to the corresponding radiation to others, due to the accumulation of radionuclides in the body. During the period of taking radioactive drugs, patients are equivalent to a mobile radioactive source, which causes certain radiation damage to medical staff, family members, and other patients, making it necessary to take isolation and protective measures (13, 14). With the environment of the radioactive ward around, there was much inconvenience for medical staff to collect vital signs and radiation doses of patients with radioactive sources. To improve occupational radiation protection, medical staff need to wear γ-ray/β-ray protective clothing before entering the ward and minimize the time in the ward, which leads to some problems, such as the lack of effective monitoring for patients and the increased psychological burden of occupational radiation injury to medical staff. Therefore, with a replaceable mobile machine, the occurrence of alike problems would be reduced.

Recently, mobile robots have been produced, and medical staff hopes the robots can be used in isolation wards. The application of mobile robots is helpful to collect patients' vital signs and radiation dose rate information and improves the efficiency of the nursing management level for patients. Nowadays, the mobile robot has passed the registration inspection of the Shanghai Medical Device Testing Institute and obtained the test report (No. ZC 2017-789, ZC 2017-790). However, there is no clinical trial to verify the effectiveness and safety of mobile robots.

The objective of this randomized clinical trial was to evaluate the effectiveness and safety of mobile robots in providing vital signs and radiation dose rate service for patients receiving Iodine-131 radiotherapy in isolation wards.

## 2. Materials and methods

### 2.1. Study design

According to the requirements of the clinical trial protocol, this clinical trial was carried out in three medical centers (Center A, Center B, and Center C) in Shanghai and Wuhan, China, from 1 April 2018 to 1 September 2018.

This is a prospective, multicenter, randomized, paired-design clinical trial. Subjects were numbered sequentially according to the admission number and randomly assigned to group A and group B according to the randomized grouping scheme, in which group A was carried out by the robot measurement first and then by the control method; for the patients who were in group B, they measured vital signs and radiation dose rate by the control method first and then by robots.

### 2.2. Equipment

**Figure 1:**
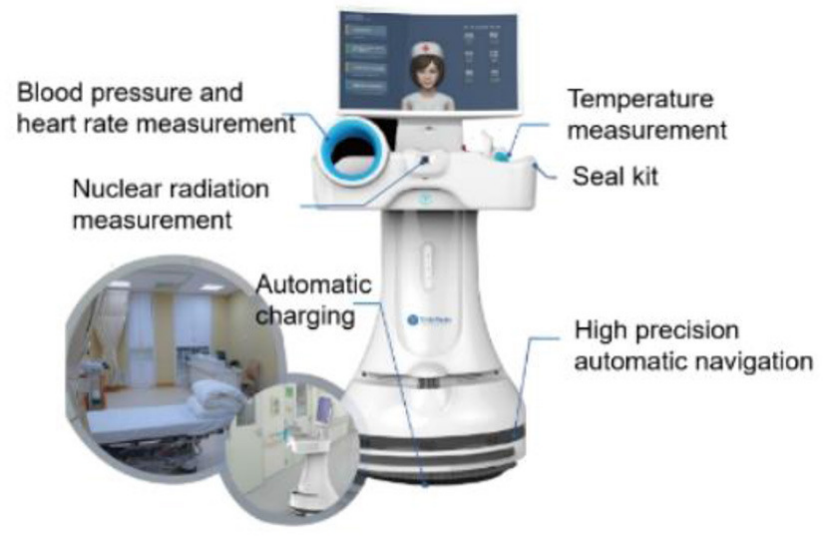
Nuclear medicine medical isolation ward mobile robot.

The instrument used in the robot measurement group was Nuclear Medicine Medical Isolation Ward Mobile Robot [mobile robot, Manufacturer: Shanghai Qinmi Robot Technology Co., Ltd., Shanghai, China (Model No: TMI-MD-NM-P)]. For product structure, the mobile robot is composed of an all-directional mobile chassis, a fuselage, a display screen, a blood pressure measurement module, a medical infrared temperature measurement module, a nuclear radiation measurement module, a charging base, and software (Figure 1).

The instruments for vital signs and radiation dose rate measurement used in the control group were an “arm automatic electronic sphygmomanometer (Manufacturer: Dongguan Fudakang Industrial Co., Ltd., Guangdong, China/Model No: FT-C25Y), infrared thermometer (Manufacturer: Dongguan Fudakang Industrial Co., Ltd., Guangdong, China/Model No: FT-F31), and nuclear radiometer (Manufacturer: (United Systems, Inc., USA/Model No: 900)” (15).

### 2.3. Matching experiment

In the paired-design experimental study, each subject would be subjected to the robot measurement and control measures in a certain order through a randomized process. A comparison trial was conducted in the morning or afternoon (within 15–24 h) on the second day after patients received radionuclide therapy (e.g., a dose of 131I drug was normally administered between 2:00 and 4:00 p.m.). Because the research results of two treatments can be obtained from the same individual and the influence of other factors can be effectively controlled, the research results are more comparable. Compared with the parallel control method, the number of subjects can be reduced, which is an effective control method.

### 2.4. Participants

Patients who were receiving radionuclide isolation therapy were selected as the target population. They were recruited from three medical centers. The recruitment was from 1 April 2018 to 1 September 2018. Follow-up was completed on 28 September 2018.

#### 2.4.1. Inclusion criteria

The inclusion criteria were the following: inpatients in a nuclear medicine isolation ward with independent ability to cooperate with researchers; adults, 18–65 years old, with no gender limitation; and volunteer to participate and sign the informed consent.

#### 2.4.2. Exclusion criteria

The exclusion criteria are the following: patients with cognitive impairment; patients with mania; deaf patients or hearing loss; patients with impaired eyesight and weak eyesight; patients with serious infectious diseases; non-cooperative or aggressive behavior (e.g., transient psychotic syndrome); patients with arrhythmia or heart disease; patients who discontinued radiopharmaceutical therapy midway; and those who are considered unsuitable to participate in the clinical trial by the researchers for other reasons.

Standards and procedures for discontinuing the radiopharmaceutical therapy: if a participant happens with serious adverse events (SAE) during the study, the ethics committee considers discontinuing the study and treatment from an ethical perspective; the study should be discontinued due to the serious impact on the clinical study for other reasons.

### 2.5. Outcome measures

The clinical trial was performed in the morning or afternoon on the 2nd day after being treated with 131I (within 36–60 h). The experimental observation contents include the success rate of the task: whether the robots complete the set task; vital sign measurement results, which include blood pressure, body temperature, and radiation dose rate; error rate of using: observe the use error of test according to the list of prediction error rate; and subjective satisfaction of patients and medical staff: the overall satisfaction evaluation of patients and medical staff was collected while the effectiveness and safety were observed. The outcome indicators include the effectiveness and safety evaluation of mobile robots.

For effectiveness evaluation, they were evaluated with the consistency between the experimental group and control group, the success rate of the task, the success rate of the first measurement as the main evaluation index, the vital sign measurement results, an error rate of use, and subjective satisfaction as the secondary indicators.

The evaluation of consistency includes blood pressure, body temperature, and radiation dose rate in the experimental group and control group. The assessment of subjective satisfaction consists of 10 items and five scales. The content of satisfaction evaluation includes the convenience of operation for temperature measurement, the convenience of operation for blood pressure measurement, the convenience of operation for residual radiation measurement, satisfaction with temperature measurement, satisfaction with blood pressure measurement, satisfaction with radiation residual measurement, the feeling for panel display, the feeling for broadcast voice, rationality and effective of the alarm and prompt, and overall satisfaction for the equipment.

Each item was rated on a 5-point scale, namely very satisfied, satisfied, average, dissatisfied, and very dissatisfied.

The evaluation of safety was evaluated by a safety assessment of the whole machine and the incidence of adverse events.

The researcher evaluates the safety of the whole machine by assessing the following 10 items of the robots: the robot fails to arrive at the task site correctly; the robot does not perform tasks according to medical instructions; the robot fails to complete the human–computer interaction with the patient; the robot fails to automatically charge properly after completing the task; whether the robot has ever collided in the process of moving; whether the robot appears the phenomenon of random walk or rotation; the robot starts to move when the subject measurement task is not finished; whether the measurement data are lost; the arm tube pressure is too high in the process of sphygmomanometer measurement, resulting in the subject's limb being squeezed; program abnormal error; unable to work, crash, or stop the machine for unknown reasons; and other.

The adverse events include the following: collision between the robot and the patients in the process of moving; the robot begins to move when the measurement task is not completely completed; during the sphygmomanometer measurement, the arm tube pressure was too high, resulting in the compression of the patient's tested limb, which in turn caused the subject to feel dizziness, headache, and even subcutaneous bleeding; and skin irritation due to contact with the test equipment.

### 2.6. Statistical analysis

The original sample size was determined to be 60 in total, which would provide 85% power, with a two-sided significance level of α = 0.05. Considering the factors of separation, the sample size was increased by 20%, and the final sample was 72, which was tested in three centers, and there were at least 16 but no more than 36 in each center.

Unless otherwise stated, analyses were performed based on the full analysis set (FAS), which is defined as the set of all randomized patients who have used the experimental method at least once. The effectiveness analysis of this study will be conducted based on the full analysis set and the per-protocol set (PPS), all baseline demographic statistics will be analyzed based on the full analysis set, and the safety evaluation will be conducted on the safety set (SS).

For effectiveness analysis, the primary endpoint (success rate of task) was evaluated using the Bland–Altman plot (16). As for secondary effectiveness indicators, they were according to the characteristics of the variables and the indicators, such as the vital sign measurement results, error rate of use, and subjective satisfaction, which were described by grouping statistics. Paired t-tests or Wilcoxon signed-rank tests were used to compare the quantitative indicators between groups. (17) Categorical data were required by matching the chi-square test (X^2^) or Fisher's exact test.

All statistical analyses were performed using SAS 9.4 statistical professional analysis software. A *p*-value ≤ 0.05 will be considered statistically significant.

### 2.7. Quality control and bias control

To avoid sampling bias and reduce the risk of spatial autocorrelation, we adopted sequential processing, namely the participants were randomly divided into group A and group B according to the test number; in group A, the participants were first measured vital signs and radiation dose rate by a mobile robot and then measured by medical staff, and in group B, the participants were first measured vital signs and radiation dose rate by medical staff and then measured by a mobile robot.

It is necessary to ensure that quality control measures are in place throughout the clinical trial process. The following specific quality control measures shall be taken: Standard operating procedure (SOP) training: Unified SOP training shall be conducted for all researchers to be familiar with the specific implementation rules and operating procedures of the clinical trial scheme and to standardize the recording methods and judgment standards.

#### 2.7.1. Clinical supervision

An independent clinical research associate (CRA) shall be set up. The CRA shall formulate the supervision plan, list, and query list and conduct on-site supervision and visits to the clinical trial institutions regularly according to the enrollment progress, so as to ensure that all contents of the study protocol are strictly followed and the study data are filled in correctly and completely. The monitoring contents include the following: check and trace all trial data case-by-case, check the integrity, authenticity, and timeliness of case report form (CRF) data records, and track and verify the adverse event (AE) report; the factors and indicators that have an important impact on the test results (including inclusion criteria, exclusion criteria, implementation scheme, effectiveness evaluation index, safety evaluation index, and shedding rate) should be verified and confirmed on-site; each CRA monitoring should provide the corresponding monitoring records and reports and submit them to the researchers; and for the problems found in the inspection, a question form should be developed and submitted to the researcher for verification and confirmation before making changes.

### 2.8. Ethics approval and informed consent

This clinical trial was conducted by the Helsinki Declaration and the Chinese Clinical Trial Research Norms and Regulations (18). This study was approved by the Chinese Ethics Committee. Participants in the trial were all given informed consent and personally signed the informed consent and dated the signing.

## 3. Results

### 3.1. Study population

**Figure 2:**
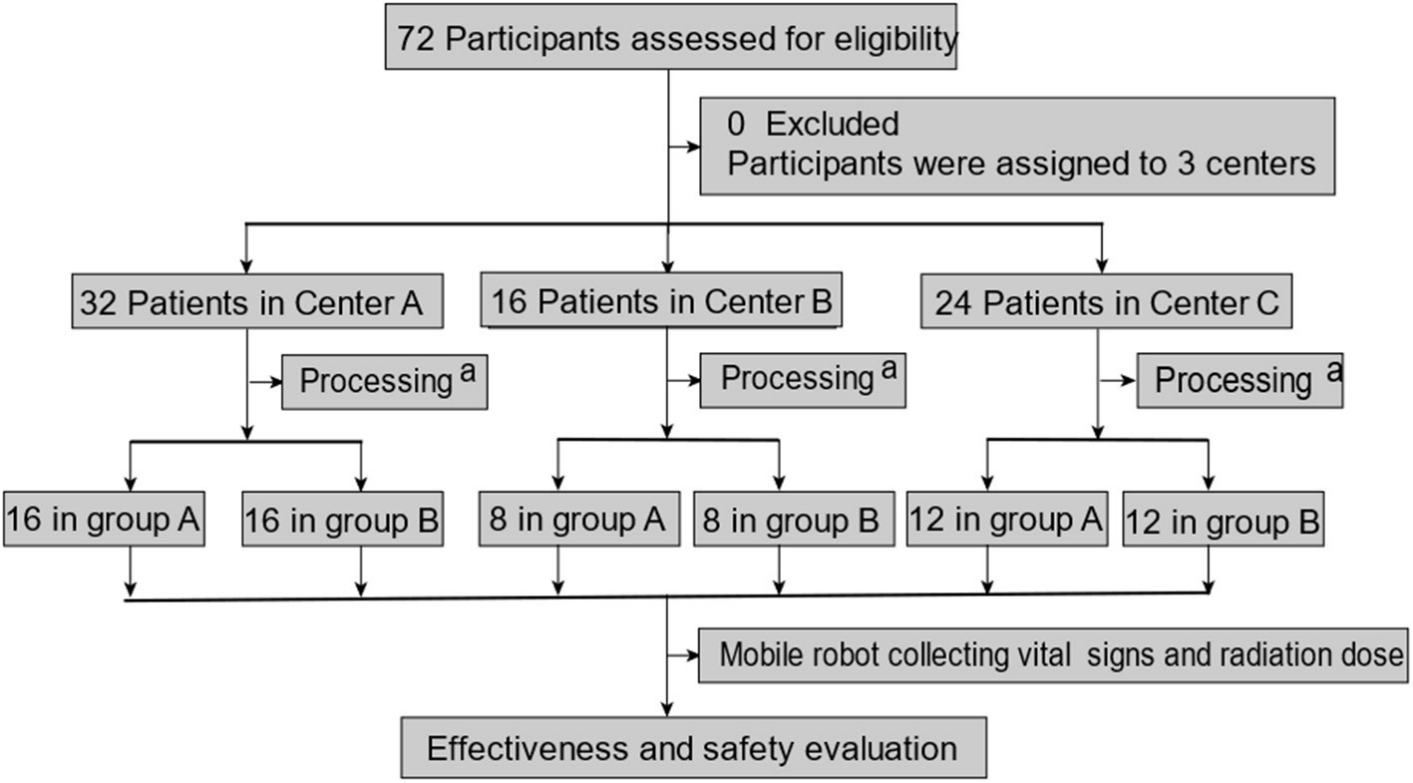
Patient enrollment and test assignment. ^a^processing includes participants being divided into two groups, and then, they were treated with 131I. A mobile robot collecting vital signs and radiation dose rate was performed in the morning or afternoon on the second day after being treated with 131I (within 15–24 h). Vital signs include temperature and blood pressure.

A total of 72 patients were enrolled in this clinical trial (32 cases from Center A, 16 cases from Center B, and 24 cases from Center C). There was no elimination or shedding case. The analysis sets of FAS, PPS, and SS included 72 cases (32 cases in Center A, 16 cases in center B, and 24 cases in center C). Our clinical trial process is shown in Figure 2; the demographic data and full analysis set are shown in Table 1.

**Table 1:** Baseline demographics and clinical characteristics of all patients with radionuclide therapy.

	**Robot measurement group (*n* = 72)**	**Control group^a^ (*n* = 72)**
**Demographic and clinical characteristics**		
Age, median (LV), y	39.5 (20–60)	39.5 (20–60)
**Sex, No. (%)**		
Male	27 (37.5)	27 (37.5)
Female	45 (62.5)	45 (62.5)
Height, median (LV), cm	164.5 (155–180)	164.5 (155–180)
Weight, median (LV), kg	62.5 (44–101)	62.5 (44–101)
The Han national, No. (%)	72 (100.0)	72 (100.0)
Allergy history, No. (%) b	4 (5.6)	4 (5.6)
**Smoking, No. (%)**		
Smoking	3 (4.2)	3 (4.2)
Gave up smoking	4 (5.6)	4 (5.6)
No smoking	65 (90.3)	65 (90.3)
**Drinking, No. (%)**		
Drinking	1 (1.4)	1 (1.4)
Gave up drinking	4 (5.7)	4 (5.7)
No drinking	67 (93.1)	67 (93.1)
Medical history, No. (%)	64 (88.9)	64 (88.9)
Surgery history, No. (%)	68 (94.4)	68 (94.4)
Coexisting diseases, No. (%)	10 (13.9)	10 (13.9)
Coexisting medication, No. (%)	5 (5.7)	5 (5.7)
**History of present illness, No. (%)**		
Post–operative isotope therapy for thyroid malignancy	67 (93.1)	67 (93.1)
Isotopic therapy for hyperthyroidism	5 (6.9)	5 (6.9)
Others	0 (0.0)	0 (0.0)
**Vital signs**		
Heart rate, median (LV), /min	78.0 (46–111)	79.5 (46–110)
Respiratory rate, median (LV), /min	18.0 (16–20)	18.0 (16–20)
Body temperature, median (LV), °C	36.5 (36.3–36.7)	36.6 (36.4–36.9)
**Blood pressure, median (LV), mmHg**		
Systolic blood pressure	114 (86–147)	116 (80–153)
Diastolic blood pressure	70.5 (53–101)	72.0 (56–109)
Radiation dose, median (LV), μSv/h	478.5 (39–1,304)	529.0 (40–1,422)

LV, limit value, including minimum and maximum values. ^a^The data of the control group and the experimental group were from the same participant, and the interval of two vital signs was 2 to 10 min. ^b^History of allergy to certain allergens, including food and medicine.

### 3.2. Effectiveness evaluation

The evaluation of effectiveness of this experiment included primary and secondary effectiveness indicators. The primary effective indicator was the consistency of the test instrument and the control instrument in measuring the completion of the task, which was evaluated by the Bland–Altman plot. In addition, the completion rates of the vital sign measurement and the success rate of the first measurement were also used as the main effectiveness measures. Secondary indicators included vital signs measurement results, the error rate of use, and subjective satisfaction.

#### 3.2.1. Primary clinical outcome

**Figure 3:**
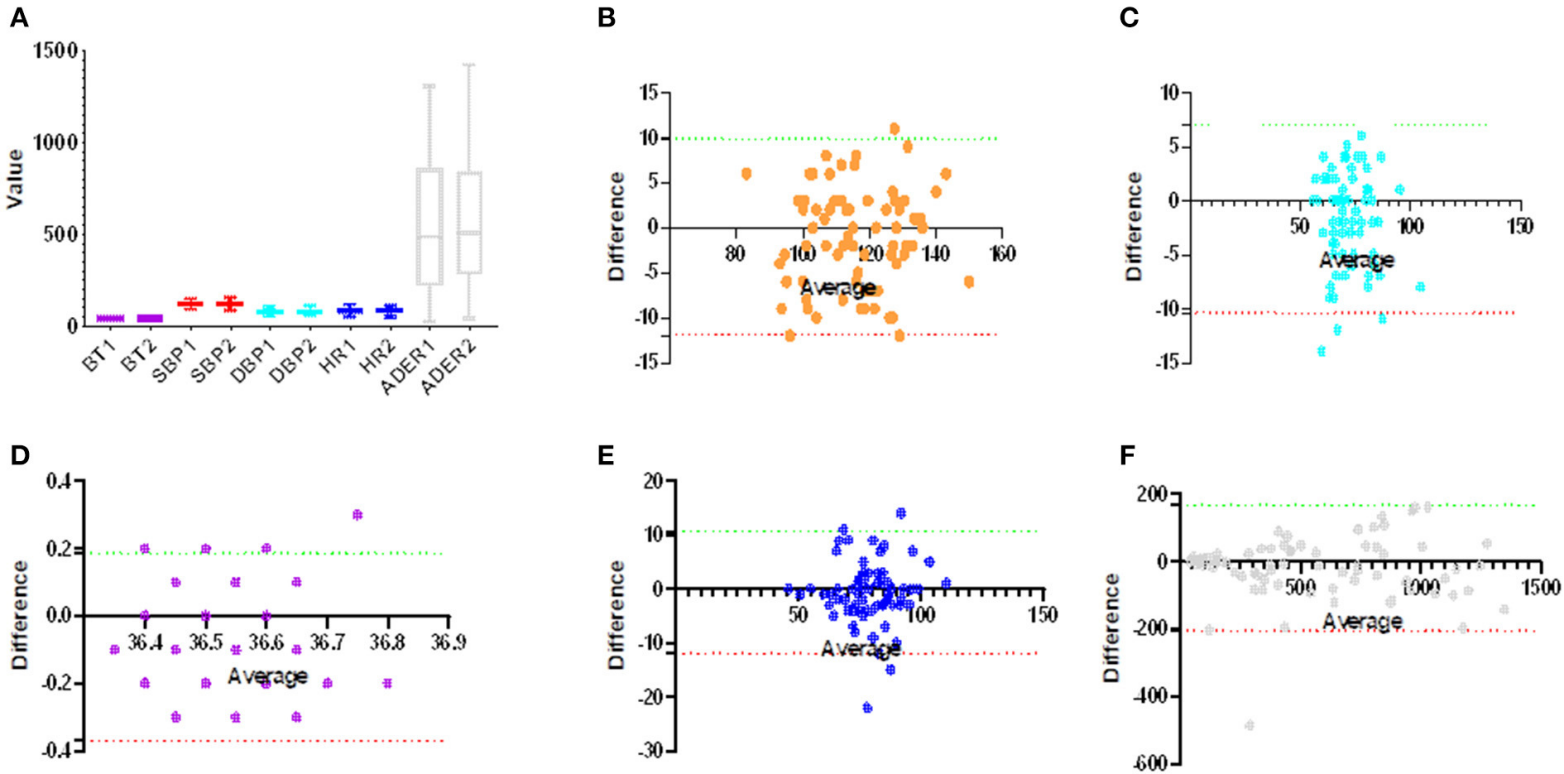
Consistency of robot measurement group and control group. **(A)** The group diagrams show the distribution of measurements for both methods, with no significant differences between the groups (*p* > 0.05). **(B–F)** A Bland–Altman plot of vital signs, **(B)** systolic blood pressure (Bias:−1.028, 95% *CI*: −11.950 and 9.897), **(C)** diastolic blood pressure (Bias: −1.708, 95% *CI*: −10.390 and 6.973), **(D)** body temperature (Bias: −0.092, 95% *CI*: −0.370 and 0.187), **(E)** heart rate (Bias: −0.667, 95% *CI*: −11.950 and 10.610), and **(F)** ambient dose equivalent rate (Bias: −19.390, 95% *CI*: −11.950 and 10.610).

The primary efficacy index of this trial was the consistency between the test instrument and the control instrument in measuring task completion. Figure 3 shows that there was relatively good agreement between the two groups in vital signs measurement, which met the design requirements. The success rate of measurement in both the experimental group and control group was good (the success rates of the first measurement of temperature, blood pressure, and radiation residue in the experimental group were 66 (91.7%), 72 (100.0%), and 72 (100.0%), and the success rates of the second or third measurement were 6 (8.3%), 0 (0.0%), and 0 (0.0%), respectively. The success rates of the first measurement of temperature, blood pressure, and radiation residue in the control group were 66 (91.7%), 68 (94.4%), and 71 (98.6%), respectively, and the success rates of the second or third measurement were 6 (8.3%), 4 (4.2%), and 1 (1.4%), respectively); there was no significant difference between experimental group and control group in the success rate of measurement (*P* = 1.000, 0.120, and 1.000). There was no significant difference between the test group and the control group in the results of collecting vital signs and radiation dose rate (*P* = 0.0001, 0.0438, and 0.0227) (Table 2).

**Table 2:** Primary clinical outcome of effectiveness evaluation.

	**Robot group (*n* = 72)**	**Control group (*n* = 72)**	**χ^2a^**	***P*-value^b^**
**Success rate of task, No./ total (%)**				
Body temperature	72/72 (100.0)	72/72 (100.0)	1.00	1.00
Blood pressure	72/72 (100.0)	72/72 (100.0)	1.00	1.00
Radiation dose	72/72 (100.0)	72/72 (100.0)	1.00	1.00
**First successful measurement** ^c^ **, No./ total (%)**				
Body temperature	66/72 (91.7)	66/72 (91.7)	1.00	1.00
Blood pressure	72/72 (100.0)	68/72 (94.4)	0.06	0.12
Radiation dose	72/72 (100.0)	71/72 (98.6)	0.50	1.00

^a^The success rate of the task was calculated by the chi-square test, and the first successful measurement was calculated by Fisher's exact test.

^b^*P*-value was calculated by the chi-square test or Fisher's exact test.

^c^The success rate of the task includes the first successful measurement and the second or third successful measurement.

#### 3.2.2. Secondary clinical outcomes

The secondary outcome indicators of this study included measurement results, the error rate of use, and subjective satisfaction.

The maximum relative deviation of the vital sign and radiation dose rate measurement results between the test instrument and the control instrument and the error rate of use were all within 10%, and there was no statistical difference between the groups (*P* = 1.000, 0.0611, and 1.000), which met the test requirements, as shown in Table 3.

**Table 3:** Secondary clinical outcome of comparison in measurement results.

	**Robot group (*n* = 72)**	**Control group (*n* = 72)**	**ICC**	**Absolute difference (95% CI)^a^**	***T*-value**	***P*-value^b^**	**MRA, %**
Body temperature, median (LV)	36.5 (36.3–36.7)	36.6 (36.4–36.9)	0.88	−0.15 (−0.17, −0.13)	−15.2	<0.001	0.46
**Blood pressure, median (LV)**							
Systolic blood pressure	114 (86–147)	116 (80–153)	0.96	−1.17(−2.17, 0.14)	−1.78	0.08	2.12
Diastolic blood pressure	70.5 (53–101)	72.0 (56–109)	0.94	−1.38(−2.40, −0.35)	−2.68	0.009	3.29
Radiation dose, median (LV)	478.5 (39–1,304)	529.0 (40–1,422)	0.99	−19.7(−36.5, −2.8)	−2.33	0.02	6.31

ICC, intra-group correlation coefficient; MRA, maximum relative deviation.

^a^The absolute differences between the experimental instrument and the control instrument were calculated based on measurement results.

^b^P-value was calculated by *t*-test.

The error rate of using each measurement index of the test equipment and control equipment was <10%, and there was no statistical difference between the test group and the control group (*P* > 0.05). The evaluation of subjective satisfaction in the robot group was very desirable, and there was no significant difference in the overall satisfaction between the experimental group and the control group (*P* > 0.05). The results of the error rate of using and subjective satisfaction are shown in Table 4.

**Table 4:** Secondary clinical outcome of the error rate of using and subjective satisfaction.

	**Robot group (*n* = 72)**	**Control group (*n* = 72)**	**χ 2/Z^a^**	***P*-value^b^**
**Error rate of using, No./total**^c^ **(%)**				
Body temperature	8/80(10.0)	7/79(8.9)	0.20	1.00
Blood pressure	0/72(0.0)	5/77(6.5)	0.04	0.06
Radiation dose	0/72(0.0)	0/72(0.0)	1.00	1.00
**Subjective satisfaction, No./total**^d^ **(%)**				
Convenience of temperature collecting	71/72(98.6)	71/72(98.6)	−0.21	0.83
Convenience of blood pressure collecting	70/72(97.2)	67/72(93.1)	−1.15	0.25
Convenience of radiation dose rate collecting	72/72(100.0)	64/72(88.9)	−2.16	0.03^*^
Satisfaction with temperature collecting	72/72(100.0)	71/72(98.6)	−1.12	0.26
Satisfaction with blood pressure collecting	69/72(95.8)	69/72(95.8)	−0.59	0.56
Satisfaction with radiation dose rate collecting	72/72(100.0)	65/72(90.3)	−2.31	0.02^*^
Feeling for panel display	70/72(97.2)	69/72(95.8)	−0.79	0.43
Feeling for broadcast voice	72/72(100.0)	69/72(95.8)	−0.53	0.60
Rationality and effective of the alarm and prompt	70/72(97.2)	67/72(93.1)	−0.74	0.46
Overall satisfaction	71/72(98.6)	67/72(93.1)	−0.74	0.46
Satisfaction with the use of medical staff ^e^	12/12(100.0)	-	-	-

^*^P < 0.05, there were differences between the two groups, and the experimental group was superior to the control group in subjective satisfaction evaluation.

^a^For error rate of using, it was calculated by the chi-square test, and for subjective satisfaction, it was calculated by Z-value.

^b^*P*-value was calculated by the chi-square test or Z-test.

^c^The total number includes 72 and the number of errors.

^d^The number of subjective satisfaction includes “very satisfied” and “satisfied,” all others were “average”.

^e^12 operators evaluated their satisfaction with the use of medical staff.

### 3.3. Safety evaluation

The safety evaluation of the mobile robot includes the researcher observing and recording all safety-related events during the functioning of the robot.

A total of 12 (13 times) adverse events (AEs) occurred during the whole study, but no serious adverse event (SAE) occurred, and all adverse events were unrelated to the study devices. The adverse event rate was 16.67%. In the whole research process, there is no safety event of the whole machine. On the whole, the mobile robot has good safety.

## 4. Discussion

In this randomized clinical trial, we demonstrated that the mobile robots had good effectiveness and safety in providing vital signs and radiation dose rate measurement service for patients receiving radionuclide therapeutic treatment. Our study found that the mobile robots had good effectiveness and safety in providing vital signs and radiation dose rate measurement service for patients, and there was no statistical difference between the experimental group and the control group.

The mobile robots had good effectiveness and safety in providing vital signs, and the radiation dose rate measurement service for patients had been demonstrated in two ways. First, there was good consistency between the two groups, and the completion rates of the vital signs measurement and the success rates of the first measurement were good. The maximum relative deviation of the vital sign and radiation dose rate measurement results between the test instrument and the control instrument and the error rates of use were all within 10%, which met the test requirements. The mobile robots gained good satisfactory evaluation, and there was no significant difference in the overall satisfaction evaluation between the experimental group and the control group. These features showed that the mobile robots had good effectiveness in providing vital signs and radiation dose rate measurement service. Second, although the adverse events were 13, there was no adverse event related to robots and the serious adverse event was 0, which indicated that the robots had good safety.

In this study, we used mobile robots in the radionuclide isolation ward to provide vital sign measurements for patients receiving Iodine-131 treatment. Unlike our study, some previous trials were conducted in general wards or other environments (10, 19–21). Our study found that the mobile robots had good effectiveness and safety, which are significant for mobile robots being used in radionuclide isolation wards. By using mobile robots, the problem of lacking effective monitoring for patients and the increasing psychological burden of occupational radiation injury to medical staff would be solved. Significantly, the global pandemic of coronavirus disease 2019 (COVID-19) began in December 2019 (22); up to now, the global form of COVID-19 is still very severe, and COVID-19 has a very strong infectivity (23). Even in the isolation wards, the medical staff cannot completely block the spread of COVID-19 (24). However, it could reduce the exposure of the medical staff to coronavirus disease infections when our mobile robots are engaged in the COVID-19 isolation wards (25). Similarly, for other Class A infectious diseases, the application of mobile robots in these isolation wards would bring a lot of conveniences and reduce the exposure risk for medical staff. Besides, the mobile robots also have other functions as shown in the equipment introduction part, which would be convenient for patients to acquire essential service and improve the working efficiency of medical staff.

Our study had several limitations. First, our study was in nuclear medicine isolation wards, and the patients were 20–60 years old with independent ability to cooperate with researchers; it is unknown whether our results are applicable to others. Second, our study concentrated on the effectiveness and safety of mobile robots; these aspects such as economics and appearance must be confirmed in another study. Third, the evaluation list of subjective satisfaction was designed by investigators, and there may be some contingency in satisfaction evaluation. It is unknown whether the mobile robots are applicable to all the patients in the nuclear medicine isolation wards. It is essential to carry out subsequent studies and feedback to improve the function of the robots.

In summary, the mobile robots have good effectiveness and safety in providing vital signs and radiation dose rate measurement service for patients receiving Iodine-131 treatment, and they could reduce the radiation to medical personnel and provide convenience for patients and medical personnel.

## 5. Conclusion

Our finding was that the mobile robots had good effectiveness and safety in providing vital signs and radiation dose rate measurement for patients receiving Iodine-131 treatment and they could be used in isolation wards.

## Data availability statement

The original contributions presented in the study are included in the article/supplementary material, further inquiries can be directed to the corresponding authors.

## Ethics statement

The studies involving human participants were reviewed and approved by the Ethics Committee of Shanghai 10th People's Hospital (Tenth People's Hospital of Tongji University). The patients/participants provided their written informed consent to participate in this study.

## Author contributions

Conceptualization: WJ. Data curation: YJ. Funding acquisition: ZL. Investigation and supervision: SF. Methodology: DL and DG. Project administration: ZG, JL, and ZL. Resources: ZG. Software: GL. Validation: XY. Visualization: MS. Writing—original draft: DG. Writing—review and editing: DL. All authors contributed to the article and approved the submitted version.
